# Stridulatory Sound-Production and Its Function in Females of the Cicada *Subpsaltria yangi*

**DOI:** 10.1371/journal.pone.0118667

**Published:** 2015-02-24

**Authors:** Changqing Luo, Cong Wei

**Affiliations:** Key Laboratory of Plant Protection Resources and Pest Management, Ministry of Education, Entomological Museum, Northwest A&F University, Yangling, Shaanxi, 712100, China; Universidad Nacional Autonoma de Mexico, MEXICO

## Abstract

Acoustic behavior plays a crucial role in many aspects of cicada biology, such as reproduction and intrasexual competition. Although female sound production has been reported in some cicada species, acoustic behavior of female cicadas has received little attention. In cicada *Subpsaltria yangi*, the females possess a pair of unusually well-developed stridulatory organs. Here, sound production and its function in females of this remarkable cicada species were investigated. We revealed that the females could produce sounds by stridulatory mechanism during pair formation, and the sounds were able to elicit both acoustic and phonotactic responses from males. In addition, the forewings would strike the body during performing stridulatory sound-producing movements, which generated impact sounds. Acoustic playback experiments indicated that the impact sounds played no role in the behavioral context of pair formation. This study provides the first experimental evidence that females of a cicada species can generate sounds by stridulatory mechanism. We anticipate that our results will promote acoustic studies on females of other cicada species which also possess stridulatory system.

## Introduction

Acoustic signaling is a widespread form of communication and occurs not only in vertebrates, but also in arthropods and even in plants [1–4]. Among animals, acoustic communication is used in different behavioral contexts, such as intra-sexual competition, inter-sexual interactions, and territorial defense [2,5,6]. For example, sound-based communication plays a vital role in reproduction in a diverse range of taxa (e.g., frogs and insects), in which acoustic advertisement is generally the domain of males [2,6]. The number and kind of communicative signals in the repertoire of an animal species depend on the complexity of its life activities [7]. Different acoustic properties of communication signals potentially encode different kinds of biologically significant information such as sex, dominance status and physical condition [8]. Sound-producing behavior has evolved in diverse arachnid and insect groups. Many species of spiders can produce sounds by stridulatory mechanism, and 12 different types of stridulatory apparatus are known in spiders [9–11]. Acoustic signals of spiders are used in inter-sexual communication, intra-sexual agonistic interactions, or to reinforce other defensive signals showed towards potential predators [12–14]. In the Insecta, sound-producing structures may involve almost any part of the insect’s exoskeleton [15], and the main sound-producing mechanisms are vibration, percussion, stridulation, clicking mechanism, and air expulsion [16,17].

Among insects, cicadas are well-known for their tymbal sound-producing mechanism in males [18]. The tymbal organ is essentially composed of a ribbed membrane at the base of the abdomen and an attached muscle, and sounds are generated when the tymbal muscle activity deforms the stiff membrane [19–21]. Male cicadas emit different types of acoustic signals in different behavioral contexts in order to gain benefits such as attracting conspecific females and deterring predators [18, 22–25].

Sound production is generally thought to be restricted to male cicadas, and most previous studies have focused on acoustic behavior of the males. However, in some cicada species, females are able to generate sounds, functioning in intersexual communication, by means of wing-flicking [26–30]. Females in these cicadas usually do not have any specialized sound-producing structures except that in some species the forewing costa is distinctly angled [31,32].

What is interesting and unusual is that females of the cicada *Subpsaltria yangi* Chen possess a pair of stridulatory organs [33]. The stridulatory organ is a file and scraper system similar to that found in crickets, katydids and grasshoppers [32–34]. *S*. *yangi* is a medium-sized cicada with adult body length of 28.0–33.2 mm, and is the only known cicada species of the subfamily Tettigadinae in China [33,35]. In the present study, we conducted scraper-ablation experiments to illustrate how females of *S*. *yangi* produce sounds. Furthermore, acoustic playback experiments were carried out to explore how males respond to sounds produced by *S*. *yangi* females, and how different components of the female sounds function in the behavioral context of pair formation.

## Materials and Methods

### Ethics Statement

No specific permits were required for this study. This study did not involve endangered or protected species, and the cicada *Subpsaltria yangi* used in the present study was not included in the “List of Protected Animals in China”.

### Study site and species

**Fig 1:**
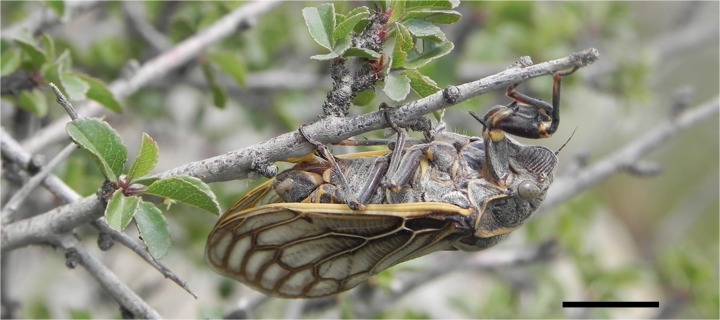
A female of *Subpsaltria yangi*. Scale bar, 1 cm.

The study site, Chunshugou valley (38°33.699′N, 105°55.217′E) where the cicada *Subpsaltria yangi* is particularly abundant (Fig. 1), is located in the Helanshan National Nature Reserve, Ningxia Hui Nationality Autonomous Region, China. This cicada population occurs at elevations between 1400 m and 1600 m. Investigations were performed between 6 and 26 June 2014. Adults of this cicada species feed mainly on *Ephedra lepidosperma* (Ephedraceae), a medicinal plant used in traditional Chinese medicine. Voucher specimens are deposited in the scientific collection of the Entomological Museum, Northwest A&F University (NWAFU), China.

### Morphology of the stridulatory organ

We examined the morphology of the scraper and the file using an Olympus SZX 10 stereomicroscope (Olympus Corporation, Tokyo, Japan). The number of ridges of the file was counted. Micrographs were captured with a Retiga 2000R digital camera (QImaging, Canada) mounted on a Nikon SMZ 1500 stereoscopic zoom microscope (Nikon Corporation, Tokyo, Japan), and then 80 sequential shots at different focal depths were processed using the Auto-Montage Pro software to generate a single composite image. The morphology of the file and the scraper were also examined using a scanning electron microscope (S-3400N, Hitachi, Tokyo, Japan).

### Sound recording and analysis

The sounds produced by *S*. *yangi* females were recorded using a linear PCM recorder with stereo microphones (PCM-D50, Sony, China; frequency range 20–20000 Hz and a 44.1 kHz/16 bit sampling resolution). The sounds were recorded in WAV file format, and the sounds recorded on the left channel of the recorder were used for acoustic analysis. Acoustic analysis was conducted using the Raven Pro 1.4 (The Cornell Lab of Ornithology, Ithaca, NY, USA) and the Seewave package [36], a custom-made library of the R software platform [37].

### Scraper-ablation experiments

**Fig 2:**
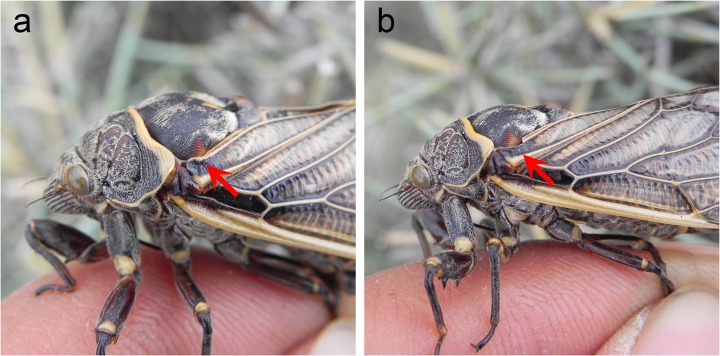
Scraper-ablation experiment. (a) The red arrow indicates one of the paired stridulatory organs of a female. (b) The red arrow indicates that the scraper of the stridulatory organ was removed.

Females of *S*. *yangi* produced sounds in response to acoustically advertising males. Behavioral observations on the sound-producing behavior of *S*. *yangi* females indicated that sounds were emitted during the rapid upward and downward movements of the forewings (S1 Video). The forewing movements might cause interaction between the scraper and the file (Fig. 2A), so the sounds were probably generated by the stridulatory mechanism. However, similar to the sound-producing mechanism of the wing-banger cicada *Platypedia putnami*, the sounds might be produced by banging the tegmina against the body [34]. Therefore, it is difficult to explicitly determine the underlying mechanism of sound production in *S*. *yangi* females. To determine the role of the stridulatory organs in sound production, we conducted scraper-ablation experiments in which sounds recorded from females with and without stridulatory scrapers were analyzed and compared.

The captured females were kept on their host plants covered with gauze netting until testing. Scraper-ablation experiments were conducted only with mature and unmated females, because our previous field work had demonstrated that only unmated mature females of this species were acoustically responding to advertising males, which is the same to periodical cicadas [28]. The captured females were tested individually. A female was placed in a small cage (0.25×0.25×0.50 m), in which the insect behaved normally according to behavioral observations. The sounds emitted by the caged female were recorded. The paired scrapers of the female were then carefully removed with surgical scissors (Fig. 2A, B). When the female performed normal sound-producing movements (i.e., upward and downward movements of the forewings), the sounds emitted by the female were recorded second time.

A total of 15 females were used in scraper-ablation experiments. All experiments were carried out between 9:00 am and 3:00 pm, a period corresponding to the peak singing activity of this cicada. During the experiments, the ambient temperature ranged from 29 to 34°C.

### Male responses to female sounds

Behavioral observations suggested that the sounds produced by *S*. *yangi* females functioned in the behavioral context of pair formation. To determine whether the sounds produced by *Subpsaltria* females elicit acoustic and phonotactic responses from conspecific males, we conducted acoustic playback experiments in the field.

A high-quality sound recording (i.e., having high amplitude relative to background noise and no overlap with other sounds) from a female of *S*. *yangi* was selected for playback experiments. Playbacks were conducted using a Sony PCM-D50 Linear PCM Recorder and a Mogic Q2 loudspeaker (frequency response, 150–20000 Hz). A digital sound level meter (Benetech GM1357; fast response, A weighting) was used to measure sound pressure levels. The peak output intensity of the loudspeaker was adjusted to 60 dB SPL measured at 50 cm from the loudspeaker. This SPL is within the range of natural variation recorded in the cicada population (range, 55.4–65.8 dB SPL; mean ± SD = 60.5 ± 3.2 dB SPL; *N* = 19). We performed experiments between 9:00 am and 3:00 pm. Vegetation in the dry habitat occupied by *S*. *yangi* consists primarily of drought-tolerant dwarf shrubs and herbaceous plants, generally not exceeding one meter in height.

We tested whether the female sounds elicit phonotactic responses from conspecific males in the field. A total of 25 males were used for this experiment. We placed the loudspeaker on the ground and about 3 m from an acoustically advertising male. Then, the acoustic stimulus was presented from the loudspeaker. The repetition rate of the sounds was 82 sounds per minute. During the playback period, a phonotactic response was noted if the male flying towards and landing within 50 cm of the loudspeaker. A ‘no response’ was noted if the male flying away or remaining still after 3 min of the playback.

We tested whether the female sounds elicit acoustic responses from conspecific males in the field. A total of 35 males were used for this experiment. A single male was placed in a cage (0.25×0.25×0.50 m). The loudspeaker was placed approximately 1 m from the male. There was a 3-min control period in which no sound was presented, followed by a 3-min test period in which the acoustic stimulus was emitted by the loudspeaker. The sound pressure level at the position of the tested male was about 55 dB SPL. During the control and test periods, we recorded whether the male produce sounds in response to the playback. The playback experiments were conducted far away from the studied cicada population to avoid the possible influence of the sounds produced by other males and females in the natural population.

### Behavioral importance of different components of female sounds

**Fig 3:**
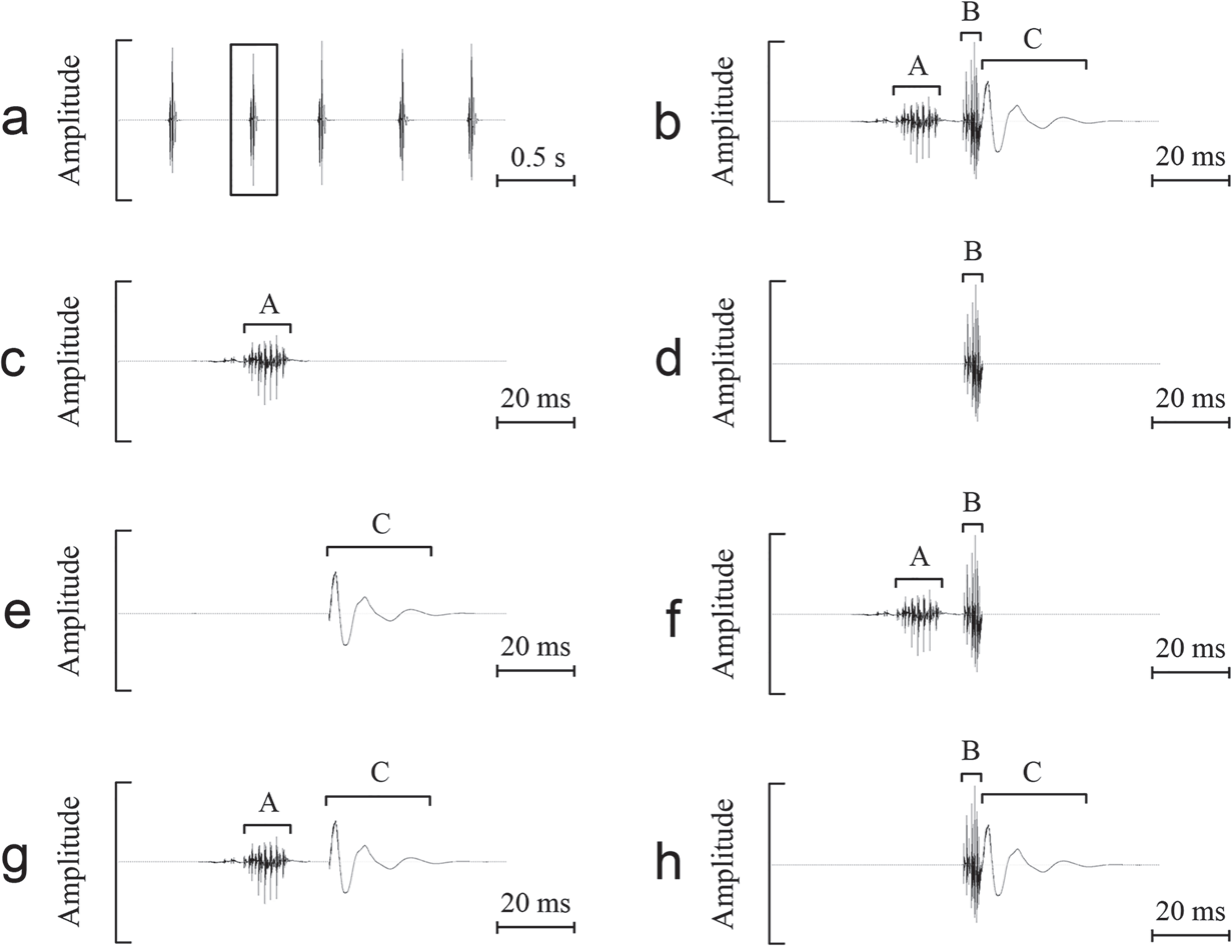
Acoustic playback stimuli. (a) Oscillogram of a part of stimulus ABC. (b) Detailed oscillogram of a female sound marked by the box in *a*. (c, d, and e) Detailed oscillograms of a modified female sound included in stimulus A, B, and C, respectively. (f, g, and h) Detailed oscillograms of a modified female sound included in stimulus AB, AC, and BC, respectively.

Acoustic playback experiments demonstrated that female sounds could stimulate acoustic and phonotactic responses from males (see *Male responses to female sounds* in Results). Acoustic analysis revealed that each female sound could be divided into three components: A, B, and C. Both component A and B were produced by stridulatory organs, whereas component C resulted from the impact between the forewings and the body (see *Scraper-ablation experiments* in Results). To explore whether only one component, or the combination of two components or all the three components play a role in eliciting male responses, further playback experiments were carried out to investigate the efficiency of various acoustic stimuli in eliciting acoustic and phonotactic responses from males. We created seven types of playback stimuli: A, B, C, AB, AC, BC, and ABC. Stimulus ABC was a natural recording of one *S*. *yangi* female (Fig. 3A, B; S1 Audio). Stimuli A, B, C, AB, AC, and BC were modified from stimulus ABC. Stimuli A, B and C were created, respectively, by replacing the component B and C, component A and C, and component A and B found in each female sound of the stimulus ABC with silence (Fig. 3C–E; S2–S4 Audios). Stimuli AB, AC and BC were created, respectively, by replacing the component C, component B, and component A found in each female sound of the stimulus ABC with silence (Fig. 3F–H; S5–S7 Audios). These acoustic stimuli were generated at a sample rate of 44.1 kHz and 16-bit resolution with the R package Seewave. The playback methods used were similar to that described above (i.e., *Male responses to female sounds*).

### Statistical analysis

Statistical analysis was undertaken with SPSS 17.0 software. Data are presented as means ± S.D. All statistical tests were two-tailed, and *P* < 0.05 was considered significant.

## Results

### Morphology of the stridulatory organ

**Fig 4:**
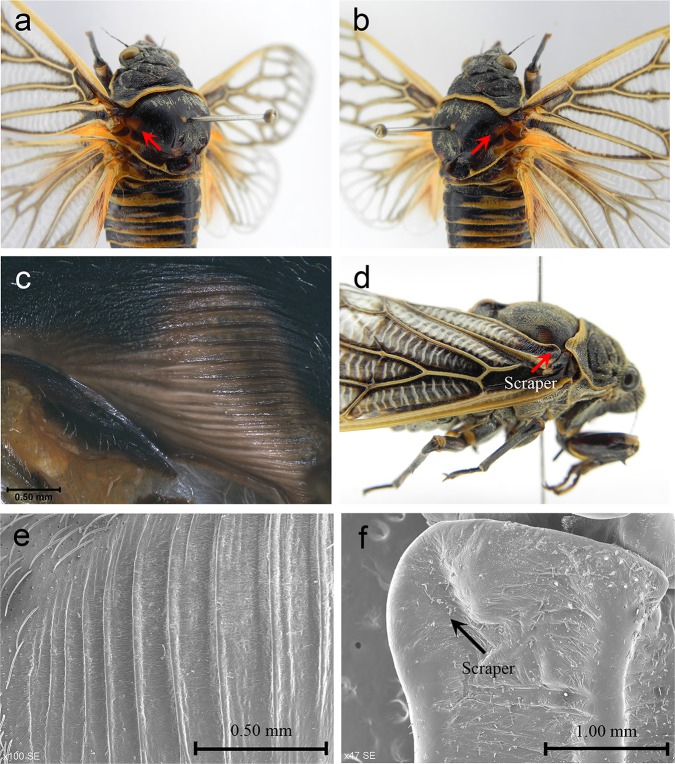
The paired stridulatory organs of a female. (a) A file (red arrow) is situated at the left anterior angle of the mesonotum. (b) A file (red arrow) is situated at the right anterior angle of the mesonotum. (c) A micrograph of a stridulatory file. (d) The red arrow indicates the scraper of a stridulatory organ. (e) A scanning electron micrograph showing the ridges of a file. (f) A scanning electron micrograph showing the scraper of a stridulatory organ.

*Subpsaltria yangi* females have a stridulatory organ on each side of the body. The left and right stridulatory organs are similar in morphology. The stridulatory file is a conspicuous oval area on the anterior angle of the mesonotum (Fig. 4A, B). The file is yellowish in colour, and bears a series of ridges (Fig. 4C). The ridges are highly sclerotized and almost parallel to each other (Fig. 4C, E). There was no significant difference between the number of ridges on left and right files (left: 17.50 ± 2.76; right: 17.90 ± 2.13; paired *t*-test, *P* > 0.05, *N* = 20). The base of the inner margin of the forewing, serving as the scraper, is thickened, sclerotized and slightly curved outwards (Fig. 4D, F).

### Scraper-ablation experiments

**Fig 5:**
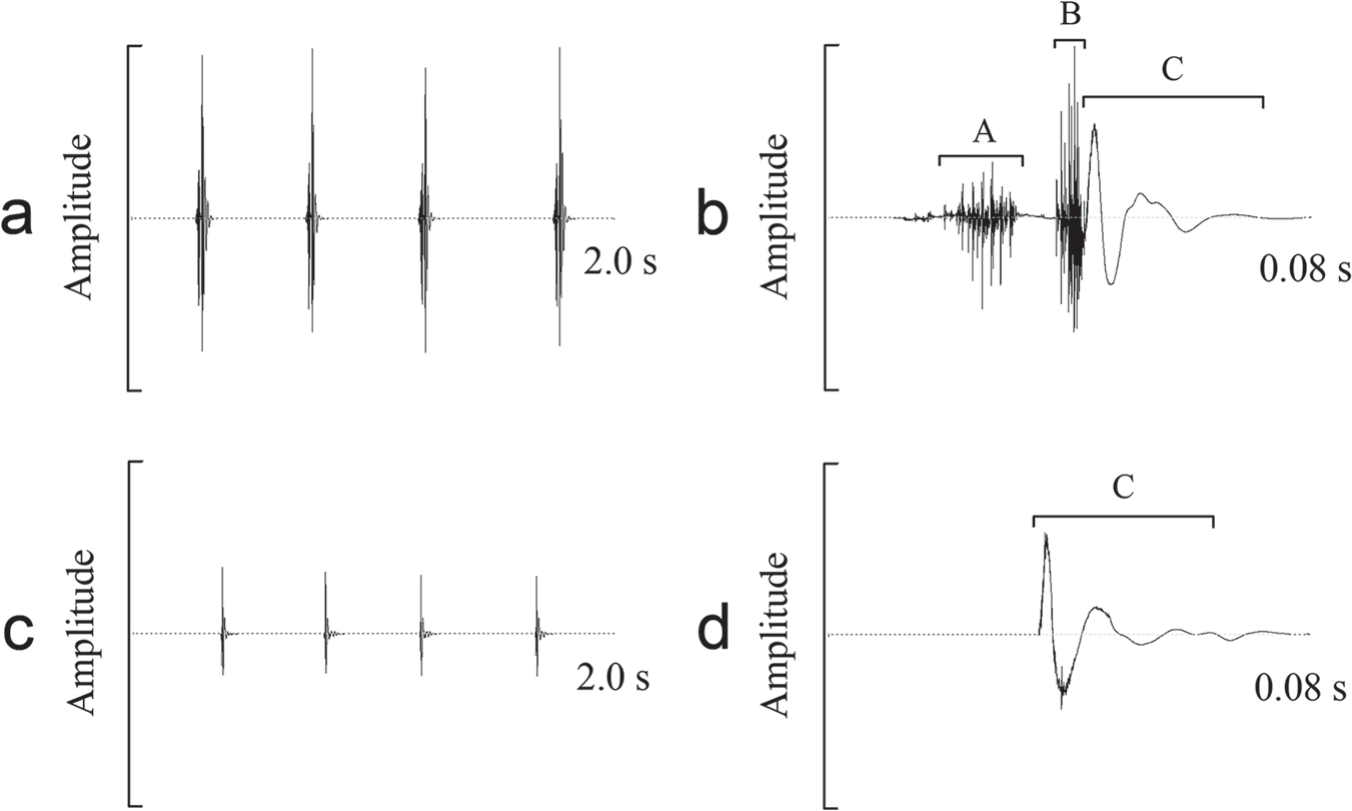
Comparison of sounds recorded from the females before and after ablation of the stridulatory scrapers. (a) Oscillogram of four sounds produced by a pre-ablated female. (b) Detailed oscillogram of a single sound produced by a pre-ablated female. (c) Oscillogram of four sounds produced by a post-ablated female. (d) Detailed oscillogram of a single sound produced by a post-ablated female.

In *S*. *yangi* females, sound production was correlated with the fast forewing movements (S1 Video). A typical female sound had a complex and stereotyped structure, and could be divided into three components: A, B, and C (Fig. 5A, B; S8 Audio). After ablation of the scrapers on the forewings, the female cicadas could normally perform the sound-producing movements (i.e., upward and downward movements of the forewings). However, the sound produced by post-ablated females did not show a three-component structure, and consisted of only component C (Fig. 5C, D). Without scrapers, the females lost their ability to produce components A and B. These results demonstrated that components A and B found in female sounds were produced by the stridulatory mechanism. An upward movement of the forewings led to a file-scraper interaction, producing the component A. The downward movement of the forewings resulted in a similar file-scraper interaction, and the component B was generated. An impact between the forewings and the body occurred when the forewings moved down to their resting position. This impact induced the production of the impact sound, i.e., component C.

### Male responses to female sounds

Playback experiments to calling males in the field showed that males of *S*. *yangi* displayed a strong phonotactic response to conspecific female sounds. All males (*N* = 25) used in the experiments flew towards the loudspeaker, from which the female sounds were broadcasted. The males landed near the loudspeaker, and some of them ultimately made physical contact with the loudspeaker. Another finding was that the female sounds were able to evoke acoustic responses from all males tested (*N* = 35).

### Behavioral importance of different components of female sounds

**Fig 6:**
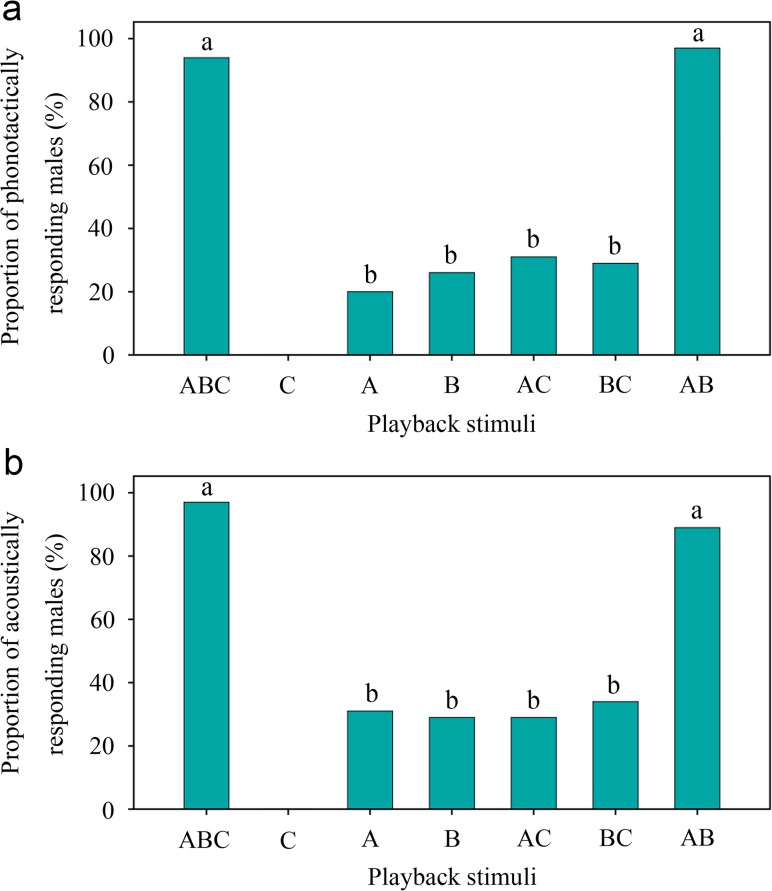
Efficiency of the seven acoustic stimuli in eliciting behavioral responses from males. (a) Males’ phonotactic responses to different types of acoustic stimuli. Different letters indicate a significant difference (*P* < 0.05). (b) Males’ acoustic responses to different types of acoustic stimuli. Different letters indicate a significant difference (*P* < 0.05).

In a different set of playback experiments, stimulus ABC (viz. natural female sounds) evoked high levels of behavioral responses from males. Thirty-three of 35 (94%) males showed phonotactic responses to stimulus ABC (Fig. 6A), and this stimulus evoked acoustic responses from 34 of 35 (97%) males (Fig. 6B). In contrast, stimulus C was unable to elicit acoustic and phonotactic responses from males (Fig. 6A, B). Both stimuli A and B could evoke acoustic and phonotactic responses from males, but they were significantly less effective than stimulus ABC (Fisher’s exact test, *P* < 0.05 in all cases; Fig. 6A, B). Similarly, stimuli AC and BC triggered significantly weaker behavioral responses from males than stimulus ABC (Fisher’s exact test, *P* < 0.05 in all cases; Fig. 6A, B). Stimulus AB was as effective as stimulus ABC in eliciting male responses (Fisher’s exact test, *P* > 0.05 in all cases; Fig. 6A, B). Altogether, these results suggest that both component A and component B produced by the stridulatory organs are essential to effectively elicit male phonotactic and acoustic responses, whereas component C is neither necessary nor sufficient to evoke behavioral responses from males.

## Discussion

In insects, stridulatory mechanism is an extremely widespread sound-producing method, and such mechanism has been described in at least seven different insect orders [17,38]. Stridulatory structures and mechanisms are particularly well understood in Orthoptera [39–41]. In cicadas, the main method of sound production is the tymbal mechanism, and the tymbal system is only developed in males, except that in two species from the family Tettigarctidae, *Tettigarcta crinita* and *T*. *tomentosa*, both sexes possess rudimentary tymbals with reduced apparent tymbal muscles [42,43]. As in most other cicada species, the females of *Subpsaltria yangi* lack tymbal organs, but they have evolved well-developed stridulatory structures. In the present study, we explored the possibility of stridulatory sound production by *S*. *yangi* females. We showed that females of *S*. *yangi* were capable of producing sounds with their stridulatory organs. This is, to our knowledge, the first experimental demonstration that females of a cicada species can emit sounds by stridulatory mechanism. Our study adds to increasing evidence that specialized sound-producing organs and acoustic signaling are not restricted to males of cicadas.

The functions of female sounds have been studied in many animal taxa. In birds, sounds are used by females to attract mates [44–46], to defend territories [47–49], to ensure male parental care [50], and to maintain intrapair contact and coordinate breeding activities [51–54]. Previous studies have shown that female anurans produce sounds to incite male-male competition [55,56], to stimulate male vocalization [57,58], and to attract males [59,60]. In spiders, females of some species produce stridulatory sound signals to induce changes in male genitalic movements during copulation [13], and to inform males of sexual receptivity [14]. In some insects, female sound production plays a role in courtship communication [61–64] and in deterring predators [65]. In this study, we investigated the functional significance of female sound production in the cicada *Subpsaltria yangi*. The female sounds are produced in response to calling songs of males. Our acoustic playback experiments clearly demonstrate that the sounds emitted by *S*. *yangi* females can elicit acoustic and phonotactic responses from conspecific males. The sounds produced by the females of this cicada species operate as intraspecific communicative signals, and function in the behavioral context of pair formation. The communication system of *S*. *yangi*, characterised by male advertisement and female acoustic response, is similar to that of the periodical cicadas *Magicicada* spp. [28] and the Australian tick-tock cicada *Cicadetta quadricincta* [27].

Scraper-ablation experiments and acoustic analysis in our study reveal that the female sounds of *S*. *yangi* consist of stridulations produced by stridulatory mechanism and impact sounds resulted from impact between forewings and body. In the wing-banger cicada *Platypedia putnami* Davis, females can also produce impact sounds by slapping the forewings against the body, and the impact sounds are used by males for species recognition during pair formation [34]. However, our acoustic playback experiments indicate that the impact sounds produced by females of *S*. *yangi* do not play a role in pair formation, and the stridulations alone are sufficient to elicit sexual responses from males. We suggest that the impact sounds are just a functionless by-product when the female *S*. *yangi* use stridulatory system to produce sounds.

The males of *S*. *yangi* rely primarily on the females’ acoustic responses to recognize and find the females. Female sounds of this species might be only produced in response to the male calling songs during pair formation, because our field observation showed mated females stopped emitting sounds to respond to advertising males. This observation suggests that, similar to females of periodical cicadas [28], females of *S*. *yangi* may mate only once or mated females are likely to be sexually unreceptive. Moreover, there is so far no clear behavioral evidence to show that the females produce sounds in any other behavioral context. However, given our limited field observations, we cannot rule out the possibility that sound production might be used by the females in other contexts, e.g., female-female interactions. Further detailed behavioral study is needed to test this possibility.

In addition to females of *S*. *yangi*, females of some other cicada species also possess stridulatory organs [30,66]. For example, a ‘tegmen-wing’ type of stridulatory organ was described by Boulard [67] for cicadas in the genus *Maroboduus* Distant. In these species, the third anal vein of the tegmen, covered with many spines, forms the scraper, and about 50 conical teeth just behind the anterior margin of the costa of the hind wing constitute the file. Recently, Moulds [32] described a stridulatory system for the Australian genus *Cyclochila* Amyot & Serville. This stridulatory system consists of a file on the underside of the lateral angle of the pronotal collar and a scraper on the base of the forewing. Unfortunately, the sound-production and associated behaviors in females of these remarkable cicada species have not yet been properly investigated. Information yielded from acoustic studies on these cicada females is of great importance for a better understanding of the evolution of female acoustic behavior in cicadas.

## Supporting Information

S1 Audio The stimulus ABC used in acoustic playback experiments. This acoustic stimulus was created digitally at a sample rate of 44.1 kHz and 16 bits resolution.
(WAV)

S2 Audio The stimulus A used in acoustic playback experiments. This acoustic stimulus was created digitally at a sample rate of 44.1 kHz and 16 bits resolution.
(WAV)

S3 Audio The stimulus B used in acoustic playback experiments. This acoustic stimulus was created digitally at a sample rate of 44.1 kHz and 16 bits resolution.
(WAV)

S4 Audio The stimulus C used in acoustic playback experiments. This acoustic stimulus was created digitally at a sample rate of 44.1 kHz and 16 bits resolution.
(WAV)

S5 Audio The stimulus AB used in acoustic playback experiments. This acoustic stimulus was created digitally at a sample rate of 44.1 kHz and 16 bits resolution.
(WAV)

S6 Audio The stimulus AC used in acoustic playback experiments. This acoustic stimulus was created digitally at a sample rate of 44.1 kHz and 16 bits resolution.
(WAV)

S7 Audio The stimulus BC used in acoustic playback experiments. This acoustic stimulus was created digitally at a sample rate of 44.1 kHz and 16 bits resolution.
(WAV)

S8 Audio The sounds of a *Subpsaltria yangi* female. The sounds were recorded at a sample rate of 44.1 kHz and 16 bits resolution.
(WAV)

S1 Video A female of *Subpsaltria yangi* emits sounds in response to the advertisement signals produced by a male. (MP4)
